# Increased Circulating Betatrophin Concentrations in Patients with Type 2 Diabetes

**DOI:** 10.1155/2014/323407

**Published:** 2014-05-22

**Authors:** Daniel Espes, Mats Martinell, Per-Ola Carlsson

**Affiliations:** ^1^Department of Medical Cell Biology, Uppsala University, Husargatan 3, P.O. Box 571, 75123 Uppsala, Sweden; ^2^Department of Medical Sciences, Uppsala University Hospital, Uppsala University, 75185 Uppsala, Sweden; ^3^Department of Public Health Care, Uppsala University, Husargatan 3, P.O. Box 564, 75122 Uppsala, Sweden

## Abstract

Betatrophin has recently been described as a key hormone to stimulate beta-cell mass expansion in response to insulin resistance and obesity in mice. The finding has generated an interest in the development of antidiabetic drugs with betatrophin as the active component. However, the circulating levels of betatrophin in patients with type 2 diabetes are not well known. Betatrophin concentrations in plasma of 27 type 2 diabetes patients and 18 gender-, age-, and BMI-matched controls were measured. Study participants were characterized with regard to BMI, waist and hip circumference, blood pressure, and fasting plasma blood lipids, creatinine, glucose, HbA1c, and C-peptide. HOMA2 indices were calculated. Betatrophin was 40% higher in patients with type 2 diabetes (893 ± 80 versus 639 ± 66 pg/mL). Betatrophin positively correlated with age in the controls and with HbA1c in the type 2 diabetes patients. All study participants were insulin resistant with mean HOMA2B IR in both groups exceeding 2 and HOMA2%S < 50%. Control individuals had impaired fasting glucose concentrations. In this report on betatrophin concentrations in type 2 diabetes and insulin resistance, elevated betatrophin levels were measured in the patients with type 2 diabetes. Future studies are clearly needed to delineate the exact role, if any, of betatrophin in regulating human beta-cell mass.

## 1. Introduction

The hormone betatrophin, primarily produced in the liver, was recently described as a key stimulator of beta-cell mass expansion in response to obesity and insulin-resistant states in mice [1]. In fact, a 17-fold increase in beta-cell proliferation was observed when the hormone was overexpressed [1]. The secreted protein was also found in human blood. Presently, the development of drugs with betatrophin as the active component is considered for the treatment of both type 1 and type 2 diabetes.

In humans, an increased beta-cell mass by approximately 50% is observed during obesity [2]. Although minimal human beta-cell replication has been observed in such autopsy studies and in a mouse model of induced insulin resistance [3], human beta cells may proliferate in response to an obesogenic environment in mice [4, 5]. In contrast, in obese humans with type 2 diabetes a 40–60% deficit in beta-cell mass when compared to BMI-matched healthy controls has been reported [6, 7]. This may merely reflect beta-cell loss by apoptosis in manifest diabetes and could also mirror a primary defect in the beta cells to adapt and expand in response to obesity and insulin resistance. The present study aimed to investigate circulating betatrophin concentrations in type 2 diabetes patients and in BMI-matched controls without diagnosed diabetes, testing the hypothesis of a betatrophin deficiency in individuals with diabetes.

## 2. Methods

The study was approved by the Regional Ethical Board of Uppsala County and conducted in accordance with the declaration of Helsinki as revised in 2000. All study participants were given oral and written information and signed a consent form prior to inclusion in the study. Patients with type 2 diabetes (*n* = 27) were identified from the Uppsala-based diabetes registry ANDiU (http://www.andiu.se) or the Swedish National Diabetes Registry (http://www.ndr.nu). The inclusion criteria were based on WHO diagnosis criteria. Only patients with glucose-lowering treatment were included. Most of the patients had metformin as monotherapy (*n* = 19) or in combination with another oral antidiabetic drug (OAD) (*n* = 3), and one patient (*n* = 1) was treated with metformin in combination with exogenous insulin. Two patients (*n* = 2) had another OAD as monotherapy and two patients (*n* = 2) exogenous insulin. Age-, gender-, and BMI-matched nondiabetic controls (*n* = 18) were recruited through advertising at a local health care center. Inclusion criteria for controls were apart from described parameters; no history of diabetes, and no first-degree relative with diabetes. All study participants were characterized with regard to regarding weight, height, waist- and hip circumference, blood pressure and family history of diabetes, for descriptive data see Table 1. Blood samples were collected after overnight fasting (minimum 10 hours). Routine lab parameters were analysed at the central laboratory at Uppsala University Hospital. Separate blood plasma was obtained in EDTA tubes by centrifugation and then directly frozen. Betatrophin levels were analysed with an ELISA (Wuhan Eiaab Science, Wuhan, China; Catalogue number. E11644h) according to the manufacturer's protocol. All samples were analysed as duplicates and samples with coefficient of variation (CV) values >15% were excluded. We have in a previous publication confirmed the reliability of obtained ELISA values with western immunoblotting with a primary antibody (Phoenix Pharmaceuticals, Phoenix, USA; WBK-051-55) [8]. Beta-cell function at steady state (%B), insulin sensitivity (%S), and insulin resistance (IR) was estimated with the updated homeostasis model assessment (HOMA2) [9],and calculated based on fasting plasma glucose and fasting plasma C-peptide with the HOMA2 Calculator v2.2 Diabetes Trials Unit, University of Oxford.

Statistical analysis was performed using SigmaPlot 12.0 and GraphPad Prism version 6.03. An unpaired two-tailed *t* test was used to compare differences between the groups. Correlations were determined by linear regression using Pearson product moment correlation. All values are given as mean ± SEM. *P* values <0.05 were considered statistically significant.

## 3. Results

Circulating levels of betatrophin were approximately 40% higher in the type 2 diabetes patients when compared to their controls (893 ± 80 versus 639 ± 66 pg/mL; *P* = 0.03), whereas there was no difference in gender distribution, age, BMI, waist-hip circumference, or waist-to-hip ratio between the two groups (Table 1). As expected, fasting plasma glucose and HbA1c were higher in the type 2 diabetes patients (glucose 8.5 ± 0.4 versus 6.2 ± 0.2 mmol/L for controls, *P* < 0.0001; HbA1c 6.8 ± 0.1% (50.6 ± 1.6 mmol/mol) versus 5.8 ± 0.1% (40.1 ± 0.8 mmol/mol) for controls, *P* < 0.0001), but control individuals fulfilled the ADA criteria for impaired fasting plasma glucose. The patients with type 2 diabetes had a lower HOMA2%B (reflecting beta-cell function at steady state) index when compared to controls (77.9 ± 8.3 versus 108.8 ± 6.0, *P* = 0.008), whereas there was no difference in HOMA2%S (reflecting insulin sensitivity) or HOMA2 IR (reflecting insulin resistance). The plasma levels of cholesterol, HDL cholesterol, and LDL cholesterol were higher in the control group. However, the number of study participants using lipid-lowering drugs (statins) was lower in the control group (22% versus 48%), although the difference was not statistically significant. For plasma triacylglycerol levels there was no difference between the two groups.

**Figure 1:**
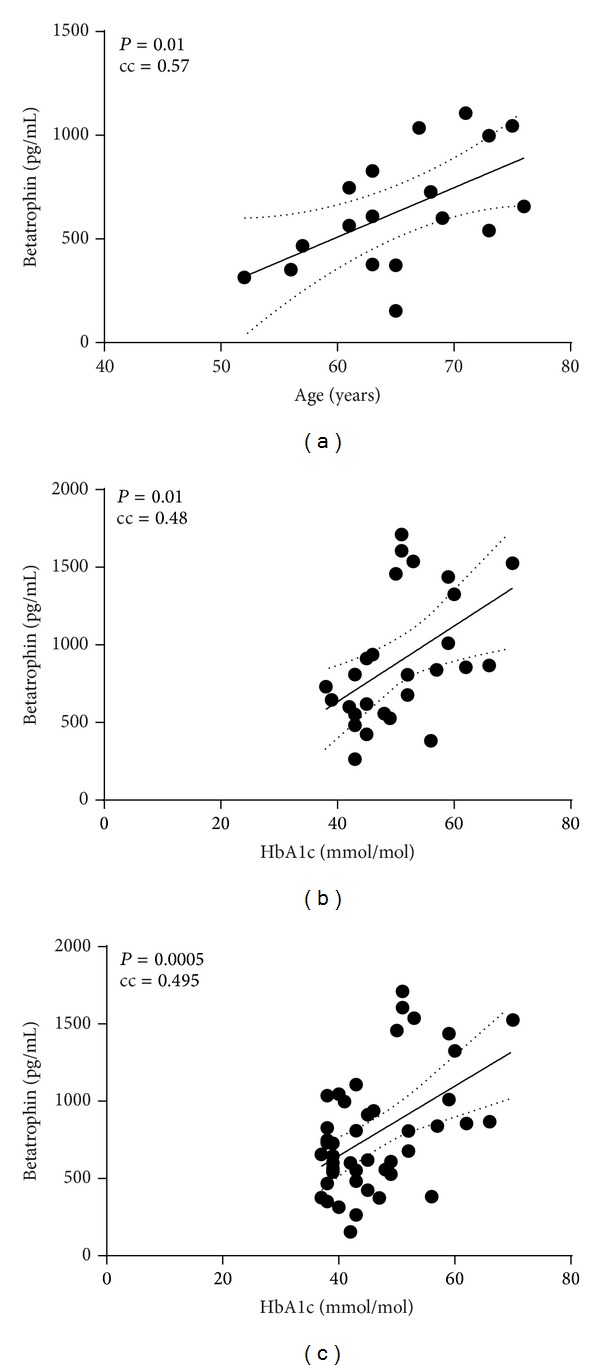
Correlation between betatrophin and age in nondiabetic controls (a) and HbA1c in patients with type 2 diabetes (b) and in all study participants (c). (a) Positive correlation between betatrophin and age in nondiabetic controls (*n* = 18, cc = 0.57, and *P* = 0.01). (b) Positive correlation between betatrophin and HbA1c for patients with type 2 diabetes (*n* = 27, cc = 0.48, and *P* = 0.01). (c) Positive correlation between betatrophin and HbA1c in all study participants (*n* = 45, cc = 0.495, and *P* = 0.0005). cc: correlation coefficient, also known as *r*. Line; best fit with 95% confidence intervals.

In controls, we observed a positive correlation between betatrophin concentrations and age (cc = 0.572, *P* = 0.01) (Figure 1(a)), whereas in the type 2 diabetes group there was no such correlation (Table 2). However, in the type 2 diabetes patients we instead observed a positive correlation between plasma betatrophin levels and HbA1c (cc 0.482, *P* = 0.01) (Figure 1(b)). There were no correlations between plasma betatrophin and other markers of metabolic control, for example, fasting plasma glucose concentrations, C-peptide concentrations, or any HOMA index, in either the controls or type 2 diabetes patients. Similarly, there were no correlations between plasma betatrophin levels and blood lipid levels in either the controls or type 2 diabetes patients. When computing correlations for all study participants, regardless of whether they were controls or patients with type 2 diabetes, we observed a positive correlation between plasma betatrophin concentrations and HbA1c (Figure 1(c)), similarly as in the type 2 diabetes group, but not with age as was observed in the control group. There was a tendency towards a positive correlation with plasma creatinine (cc = 0.267, *P* = 0.06) but not with the glomerular filtration rate (GFR) calculated with MDRD based on creatinine levels (cc = −0.208, *P* = 0.170). There was also a tendency towards a negative correlation between plasma betatrophin and plasma cholesterol (cc = −0.286, *P* = 0.06), when computing correlations for all study participants.

We therefore subanalysed the study participants with regard to whether they were treated with lipid-lowering drugs (Table 3). Among the controls, only four individuals were treated with lipid-lowering drugs, and when comparing them to controls without treatment (*n* = 14), we observed no statistical difference with regard to plasma betatrophin, cholesterol, HDL cholesterol, LDL cholesterol, triacylglycerols, or BMI. Among the patients with type 2 diabetes, there were 14 patients that were treated with lipid-lowering drugs and 13 that were not. Plasma LDL cholesterol was lower in the subjects treated with lipid-lowering drugs, whereas there was no difference for plasma betatrophin, cholesterol, HDL cholesterol, triacylglycerols, HbA1c, fasting plasma glucose, or BMI.

## 4. Discussion

The present work shows that plasma betatrophin concentrations in patients with type 2 diabetes are not subnormal, instead higher concentrations than in nondiabetic individuals were recorded. Therefore, although resistance to betatrophin effects in type 2 diabetes patients cannot be excluded, there is at least no obvious betatrophin deficiency to substitute in these individuals.

We have previously reported on betatrophin concentrations in young adult healthy controls and patients with type 1 diabetes [8]. The presently recorded betatrophin concentrations in controls were approximately doubled when compared to those in the previous study. However, at least in mice, betatrophin expression has been shown to be primarily regulated by insulin resistance in liver [1]. The controls in the previous study had a mean BMI of 23, whereas it was 29 kg/m^2^ in the present study. Moreover, the present controls had a mean waist circumference exceeding 100 cm and mean HOMA2 IR values well exceeding the 75 percentile (HOMA2 IR 1.133) of a normal Scandinavian population [10]. Also when insulin sensitivity was calculated as HOMA2%S values, these values were in the present controls only half of those in a normal Caucasian population [11]. Thus, the assigned control group in the present study was clearly insulin resistant similar to the patients with type 2 diabetes but had not developed similar decrease in beta-cell function (as assessed by HOMA2%B), despite fulfilling the ADA criteria for impaired fasting glucose. Although no strict correlations were observed between HOMA2 indices and plasma betatrophin levels, the higher betatrophin concentrations observed in the present study suggest that betatrophin expression also in humans may be induced by insulin resistance. A limitation with the present study was that insulin resistance was not measured by a glucose-clamp technique but instead estimated by HOMA2 IR indices. Nevertheless, such indices are generally considered to preferentially reflect insulin resistance in liver rather than overall insulin resistance. In this study, most of the patients were treated with metformin, which is considered as first line of treatment according to ADA and EASD position statement [12]. Therefore, very few patients with diagnosed type 2 diabetes were identified, who were treated with other antidiabetic drugs or only had diet and exercise as treatment. Theoretically, treatment with metformin could decrease plasma betatrophin levels, since it reduces insulin resistance which is described as the main stimulus for betatrophin secretion. In four identified patients without metformin there was in fact a tendency towards increased betatrophin levels when compared to those treated with metformin (1241 ± 167 (*n* = 4) versus 832 ± 85 (*n* = 23) pg/mL, *P* = 0.0694). However, since the number of patients without metformin treatment is limited this would have to be further investigated.

Our correlation analysis also identified that the plasma betatrophin concentrations in nondiabetic humans increase with age. Moreover, when including the type 2 diabetes patients with variable metabolic control, plasma betatrophin concentrations were observed to increase with HbA1c. Noteworthy, there also tended to be a positive correlation between plasma betatrophin concentrations and plasma creatinine, which would suggest that betatrophin normally is excreted in the urine, although there was no correlation between calculated GFR and betatrophin. Increased circulating concentrations of betatrophin may both reflect increased secretion and reduced clearance of the hormone, but there were no differences in plasma creatinine or calculated GFR between the type 2 diabetes patients and their controls. Betatrophin belongs to the family of angiopoietin-like proteins and has besides betatrophin been given many different names: lipasin, hepatocellular carcinoma-associated protein-TD26, RIFL, and angiopoietin-like protein 8 [13–16]. Overexpression of betatrophin in mice leads to an increase in serum triacylglycerol, and human genome-wide association studies have also shown that variations in the gene are linked with blood lipid levels [13]. In the present study, there tended to be a negative correlation between betatrophin concentrations and total plasma cholesterol levels. However, the association between betatrophin and lipid values was difficult to interpret, since several of the study patients, especially among the patients with diabetes, were treated with lipid-lowering drugs (statins). Indeed, total plasma cholesterol, LDL, and HDL were all decreased in the type 2 diabetes patients when compared to the controls. Since this study only included 45 patients, any subanalysis of betatrophin concentrations in patients with or without lipid treatment was difficult to perform with enough statistical power. Nevertheless, there seemed to be no clear difference in plasma betatrophin concentrations between statin and nonstatin treated individuals.

During the preparation of this paper, Fenzl et al. [17] reported no difference in plasma betatrophin levels between type 2 diabetes patients and nondiabetic controls in a retrospective study of stored plasma samples. This finding contradicts with the present results and previous findings on betatrophin gene expression in insulin-resistant mice [1]. The reasons for the different results obtained are presently obscure, and any potential influence of long-term or variable storage time or occurrence of any repeated freeze-thaw cycles of samples was not reported. Nevertheless, although their results showed no difference between nondiabetic and type 2 diabetic patients, there was, similar to in our study, at least no deficiency of betatrophin in the diabetic state.

We conclude that plasma betatrophin concentrations are increased in type 2 diabetes patients when compared to age-, gender-, and BMI-matched controls with similar degree of insulin resistance. Therefore, there is no obvious betatrophin deficiency to substitute in these diabetic individuals, and similar to our previous study in type 1 diabetes patients the increased plasma betatrophin concentrations seem insufficient to compensate for the development of disease by triggering a beta-cell mass expansion. Future studies are clearly needed to delineate the exact role, if any, for betatrophin in regulating human beta-cell mass.

## Figures and Tables

**Table 1:** Descriptive data and blood sample data for nondiabetic controls and patients with type 2 diabetes. All blood samples were collected after overnight fasting. HbA1c levels are given as NGSP (%) and as IFCC values (mmol/mol) in parenthesis.

Variable	Nondiabetic (*n* = 18)	Type 2 diabetes (*n* = 27)
Gender	9 male (50%)	17 male (63%)
Age (years)	65.4 ± 1.6	61.9 ± 1.7
Weight (kg)	88.6 ± 5.4	89.7 ± 3.7
BMI (kg/m^2^)	29.0 ± 1.3	30.1 ± 1.2
Waist (cm)	101.8 ± 3.8	104.3 ± 3.0
Hip (cm)	108.2 ± 2.9	111 ± 3.2
Waist-hip ratio	0.94 ± 0.02	0.97 ± 0.02
Fasting plasma glucose (mmol/L)	6.2 ± 0.2	8.5 ± 0.4*
HbA1c (% (mmol/mol))	5.8 ± 0.1% (40.1 ± 0.8)	6.8 ± 0.1%* (50.6 ± 1.6)
HOMA2%B	108.8 ± 5.95	77.9 ± 8.3*
HOMA2%S	49.1 ± 4.1	48.7 ± 7.3
HOMA2 IR	2.3 ± 0.2	3.1 ± 0.4
Fasting plasma cholesterol (mmol/L)	6.18 ± 0.27	4.55 ± 0.18*
Fasting plasma HDL cholesterol (mmol/L)	1.46 ± 0.09	1.18 ± 0.05*
Fasting plasma LDL cholesterol (mmol/L)	3.98 ± 0.22	2.80 ± 0.15*
Fasting plasma triacylglycerols (mmol/L)	1.54 ± 0.16	1.45 ± 0.12
Plasma creatinine (µmol/L)	75 ± 3	81 ± 3
MDRD-GFR (mL/min)	80.7 ± 3.3	78.4 ± 4.2
Fasting plasma C-peptide (nmol/L)	0.98 ± 0.08	1.20 ± 0.14
Fasting plasma betatrophin (pg/mL)	639 ± 66	893 ± 80*
Lipid-lowering drugs (statins)	*n* = 4 (22%)	*n* = 13 (48%)
Hypertensive treatment	*n* = 5 (28%)	*n* = 17 (63%)*

*P < 0.05.

**Table 2:** Correlations between betatrophin levels and other variables in all study participants, nondiabetic controls, and patients with type 2 diabetes. Correlations were computed with Pearson product moment.

Betatrophin correlations	All study participants (*n* = 45)	Nondiabetic (*n* = 18)	Type 2 diabetes (*n* = 27)
Age (years)	Ns, cc = 0.113	*P = 0.01 **cc = 0.572**	Ns, cc = 0.075
Weight (kg)	Ns, cc = 0.0535	Ns, cc = −0.236	Ns, cc = 0.176
BMI (kg/m^2^)	Ns, cc = 0.0915	Ns, cc = −0.164	Ns, cc = 0.15
Waist (cm)	Ns, cc = 0.111	Ns, cc = −0.173	Ns, cc = 0.192
Hip (cm)	Ns, cc = −0.0740	Ns, cc = −0.142	Ns, cc = 0.411
Waist-hip ratio	NS, cc = −0.243	Ns, cc = −0.143	Ns, cc = −0.415
Fasting plasma glucose (mmol/L)	Ns, cc = 0.235	Ns, cc = −0.054	Ns, cc = 0.082
HbA1c (% (mmol/mol))	*P = 0.0005 **cc = 0.495**	Ns, cc = −0.060	*P = 0.01 **cc = 0.482**
HOMA2%B	Ns, cc = −0.0502	Ns, cc = 0.28	Ns, cc = 0.0389
HOMA2%S	Ns, cc = 0.0685	Ns, cc = −0.13	Ns, cc = 0.119
HOMA2 IR	Ns, cc = 0.190	Ns, cc = 0.18	Ns, cc = 0.109
Fasting plasma cholesterol (mmol/L)	Ns, cc = −0.286	Ns, cc = −0.21	Ns, cc = −0.06
Fasting plasma HDL cholesterol mmol/L	Ns, cc = −0.0835	Ns, cc = 0.18	Ns, cc = −0.022
Fasting plasma LDL cholesterol mmol/L	Ns, cc = −0.230	Ns, cc = −0.33	Ns, cc = 0.087
Fasting plasma triacylglycerols (mmol/L)	Ns, cc = −0.178	Ns, cc = −0.27	Ns, cc = −0.12
Plasma creatinine (µmol/L)	Ns (*P* = 0.0579), cc = 0.267	Ns, cc = −0.045	Ns, cc = 0.342
GFR-MDRD (mL/min)	Ns (*P* = 0.17), cc = −0.208	Ns, cc = −0.169	Ns, cc = −0.209
Fasting plasma C-peptide (nmol/L)	Ns, cc = 0.168	Ns, cc = 0.195	Ns, cc = 0.103

*P < 0.05, Ns: not significant (*P* > 0.05), and cc: correlation coefficient, also known as *r*.

**Table 3:** Descriptive data and blood sample data for nondiabetic controls and patients with type 2 diabetes with or without statin treatment. Comparisons were made within groups, that is, nondiabetic controls with statin treatment versus nondiabetic controls without statin treatment and type 2 diabetes patients with statin treatment versus type 2 diabetes patients without statin treatment. HbA1c levels are given as NGSP (%) with IFCC values (mmol/mol) in parenthesis.

Variable	Nondiabetic statins (*n* = 4)	Nondiabetic no statins (*n* = 14)	Type 2 diabetes statins (*n* = 14)	Type 2 diabetes no statins (*n* = 13)
Age (years)	64.8 ± 2.7	65.6 ± 1.9	61.3 ± 2.1	62.6 ± 2.9
Weight (kg)	81.1 ± 9.7	90.9 ± 6.4	84.3 ± 4.8	95.4 ± 2.4
BMI (kg/m^2^)	27.3 ± 1.8	29.5 ± 1.7	29.0 ± 1.7	31.4 ± 1.6
Waist (cm)	96.2 ± 7.8	103.7 ± 4.4	102.0 ± 4.6	107.0 ± 3.8
Hip (cm)	105.4 ± 4.7	109.1 ± 3.6	110.5 ± 4.7	112.3 ± 3.8
Waist-hip ratio	0.91 ± 0.05	0.87 ± 0.07	0.96 ± 0.03	0.98 ± 0.04
Fasting plasma cholesterol (mmol/L)	6.25 ± 0.79	6.16 ± 0.29	4.30 ± 0.27	4.85 ± 0.20
Fasting plasma HDL cholesterol (mmol/L)	1.35 ± 0.18	1.49 ± 0.11	1.16 ± 0.08	1.19 ± 0.06
Fasting plasma LDL cholesterol (mmol/L)	4.10 ± 0.68	3.95 ± 0.23	2.44 ± 0.23	3.16 ± 0.14*
Fasting plasma triacylglycerols (mmol/L)	1.82 ± 0.39	1.46 ± 0.17	1.52 ± 0.22	1.37 ± 0.10
Fasting plasma betatrophin (pg/mL)	542 ± 77	666 ± 82	858 ± 102	930 ± 130
HbA1c (% (mmol/mol))	5.9 ± 0.2 (40.8 ± 2.1)	5.8 ± 0.1 (39.9 ± 0.8)	6.8 ± 0.2 (50.9 ± 2.5)	6.7 ± 0.2 (50.4 ± 2.1)
Fasting plasma glucose mmol/L	6.7 ± 0.4	6.0 ± 0.2	8.5 ± 0.6	8.5 ± 0.5

*P < 0.05.
